# Gaining Insights into Key Structural Hotspots within the Allosteric Binding Pockets of Protein Kinases

**DOI:** 10.3390/ijms25094725

**Published:** 2024-04-26

**Authors:** Swapnil P. Bhujbal, Joonhong Jun, Haebeen Park, Jihyun Moon, Kyungbae Min, Jung-Mi Hah

**Affiliations:** 1 College of Pharmacy, Hanyang University, Ansan 426-791, Republic of Korea; swapnil18@hanyang.ac.kr (S.P.B.); jjh0328@hanyang.ac.kr (J.J.); ddonuga@hanyang.ac.kr (H.P.); jhmoon1996@hanyang.ac.kr (J.M.); miju103@naver.com (K.M.); 2 Institute of Pharmaceutical Science and Technology, Hanyang University, Ansan 426-791, Republic of Korea

**Keywords:** protein kinase, cancer, allosteric, type III inhibitors

## Abstract

Protein kinases are essential regulators of cell function and represent one of the largest and most diverse protein families. They are particularly influential in signal transduction and coordinating complex processes like the cell cycle. Out of the 518 human protein kinases identified, 478 are part of a single superfamily sharing catalytic domains that are related in sequence. The dysregulation of protein kinases due to certain mutations has been associated with various diseases, including cancer. Although most of the protein kinase inhibitors identified as type I or type II primarily target the ATP-binding pockets of kinases, the structural and sequential resemblances among these pockets pose a significant challenge for selective inhibition. Therefore, targeting allosteric pockets that are beside highly conserved ATP pockets has emerged as a promising strategy to prevail current limitations, such as poor selectivity and drug resistance. In this article, we compared the binding pockets of various protein kinases for which allosteric (type III) inhibitors have already been developed. Additionally, understanding the structure and shape of existing ligands could aid in identifying key interaction sites within the allosteric pockets of kinases. This comprehensive review aims to facilitate the design of more effective and selective allosteric inhibitors.

## 1. Introduction

Protein kinases are part of the phosphotransferases superfamily of enzymes and are pivotal in cellular activation processes. A crucial aspect of activation involves establishing precise controls to regulate function effectively. These kinases are integral to nearly all cellular processes, including cell division, translation, transcription, various metabolic processes, and apoptosis as well. The structure of domains of protein kinase consists of two primary lobes. The first lobe is a small N-terminal, characterized by an α-helix (C-helix) and five β-sheets crucial for directing ATP binding. The second is a larger C-terminal lobe, composed of six α-helices facilitating phosphorylation and protein-substrate binding. The active site, housing the activation and catalytic loops responsible for ATP and substrate binding, is situated between these two lobes [1]. Protein kinases are categorized as either protein serine/threonine kinases or protein tyrosine kinases, depending on the nature of the phosphorylated OH group [2].

The human kinome comprises 518 protein kinases and approximately 106 pseudo kinases [3], of which, 218 kinases are implicated in human diseases. Understanding the significance of protein kinases in disease pathophysiology stems from the recognition that mutations and alterations in these kinases can disrupt cellular function, contributing to various diseases. Deregulated protein kinases are frequently observed in oncogenic cells, playing pivotal roles in their survival and proliferation [4]. With hundreds of kinases being intricately involved in cell transformation, tumor initiation, and tumor survival and proliferation [5], it has become imperative to catalog kinases within the human body to identify potential targets for novel oncology treatments. This comprehensive understanding enables the identification and treatment of abnormalities that may lead to cancer and other disorders [1].

Motivated by the favorable outcomes observed with small-molecule protein kinase inhibitors in clinical scenarios, these kinases have become a focal point of extensive research as crucial therapeutic targets in drug discovery [6]. This emphasis is notably prominent when addressing different forms of human cancers [7]. Concurrently, protein kinases have emerged as promising therapeutic targets for the treatment of cardiovascular diseases, inflammatory diseases, diabetes, as well as disorders like Parkinson’s and Alzheimer’s disease [3]. However, this article aims to review recent (United States Food and Drug Administration) FDA-approved allosteric (type III) inhibitors, elucidating their inhibition mechanisms and the distinctive characteristics of the targeted kinase binding pocket and ligand shape. These insights are crucial for the design of novel allosteric drugs.

## 2. Types of Inhibitors

Small-molecule protein kinase inhibitors are highly valuable targeted therapeutics for treating several human diseases, particularly cancers. Dar and Shokat classified these inhibitors into three categories, designated as types I, II, and III [8]. They described type I inhibitors as small molecules that bind to the active conformation within the ATP pocket of a kinase, type II inhibitors as those binding to an inactive conformation of a kinase (usually DFG-out), and type III inhibitors as allosteric or non-ATP competitive inhibitors. Allosteric compounds, which bind to a site away from the active site [9], specifically refer to compounds that bind to the exterior of the pocket where ATP typically binds in the case of kinases [2].

Gavrin and Saiah categorized allosteric inhibitors into two types (III and IV). Type III inhibitors bind in the middle of the small and large lobes adjoining to the pocket where ATP binds, while type IV inhibitors bind outside of these lobes [10]. Type III inhibitors inhabit a space adjacent to the ATP-binding pocket, allowing their simultaneous binding with ATP to the protein. These compounds act as robust-state uncompetitive or noncompetitive inhibitors concerning ATP, since ATP cannot hinder their interaction with the protein [2].

Lately, the uncovering of perspectives on the structural traits of protein kinases, their impact on enzyme activity, as well as their involvement in leading phosphorylation and substrate recognition has spurred research endeavors aimed at designing protein kinase inhibitors that avoid interfering with the ATP-binding site, which is extensively conserved. For instance, with the development of type III protein kinase inhibitors, such innovations target the identification of drugs with decreased promiscuity and associated toxicities, while also aiming to circumvent the emergence of ATP-binding-site gatekeeper mutations commonly implicated in acquired resistance to type I and II protein kinase inhibitors [3].

## 3. The Importance and Need for Allosteric Inhibitors

Although most preclinically developed small-molecule inhibitors approved for use are classified into type I and type II inhibitors, which focus on the protein kinase pocket where typically ATP binds, notable similarities in the sequential and structural characteristics of ATP pockets make achieving selective kinase inhibition quite challenging. As a result, a focus on allosteric pockets of protein kinases located exterior to the vastly conserved ATP pocket was proposed as an encouraging substitute to address existing challenges associated with kinase inhibitors, such as limited selectivity and the development of drug resistance [3].

The relatively lower sequence homology observed in allosteric sites presents distinctive opportunities for achieving more precise inhibition with minimal off-target pharmacology [11]. Allosteric inhibitors offer several advantages over traditional ATP-competitive type I and II inhibitors. They have the ability to circumvent drug resistance linked to mutations, particularly those occurring in the ATP binding site that render many ATP-competitive inhibitors ineffective, for example, the commonly encountered mutation in the important residue of Abl kinase (gatekeeper residue; T315I) [12]. Moreover, allosteric inhibitors may not require high affinity in the nanomolar range to contend with the abundant intracellular ATP concentrations, hence facilitating the recognition of weak binding inhibitors ranging from fragments to hit and lead compounds. Additionally, their potential extends beyond cancer treatment to other indications. However, numerous reported protein kinase inhibitors, including approved medications, exhibit undesired selectivity profiles [13]. Such inhibitors with a lack of specificity for the intended kinase as a drug target and limited selectivity for kinases with similar structures could result in undesirable outcomes and off-target toxicity in clinical scenarios. Furthermore, allosteric inhibitors can serve as meticulous chemical probes for advancing mechanistic investigations on molecular function. Due to these appealing attributes, allosteric inhibitors are undergoing extensive research as a novel class of small-molecule protein kinase inhibitors [3].

### FDA-Approved Allosteric (Type III) Inhibitors

Despite the relatively limited number of identified allosteric inhibitors compared to those targeting the ATP pocket, the domain of allosteric kinase inhibition has experienced rapid progress over recent years. This progress was marked by the FDA’s acceptance of trametinib as the first (type III) allosteric kinase inhibitor in 2013. Moreover, the past decade has witnessed the development of over 10 additional type III inhibitors undergoing clinical trials, along with the occurrence of a pipeline revealing potent and vastly selective preclinical drugs [3]. Several MEK1/2 (mitogen-activated protein kinase kinase) inhibitors are presently under clinical investigation for various cancers, including melanoma, acute myelogenous leukemia, gynecologic malignancies, and colorectal cancer. Trametinib has received acceptance for use, either alone or combined with the dabrafenib, which is a BRAF inhibitor, for the treatment of progressive metastatic melanoma carrying a mutation (V600E) [4,5,14,15].

**Figure 1:**
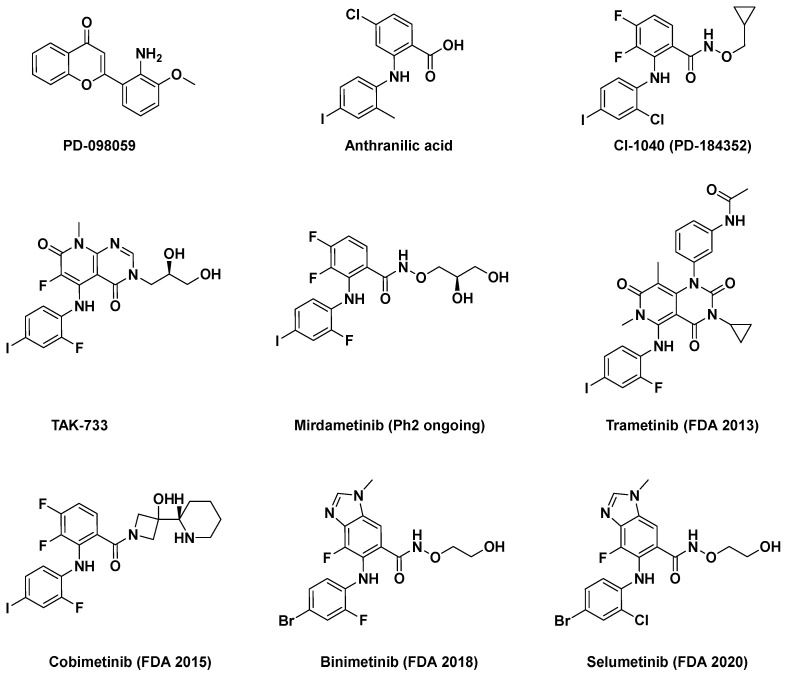
Some of the MEK1 inhibitors, including those approved by the FDA.

MEK and Akt (protein kinase B (PKB)), types of serine/threonine kinases, have been extensively studied targets for which allosteric inhibitors have been developed. Peng Wu et al. already reported comprehensive details about allosteric inhibitors targeting various protein kinases, including their structures and inhibition mechanisms, in 2015 [3]. At present, the FDA has approved four MEK inhibitors: trametinib, binimetinib, selumetinib, and cobimetinib [3,16]. Figure 1 shows some of the initially studied and FDA-approved MEK1 inhibitors. TAK-733, an investigational MEK1/2 allosteric inhibitor, is orally available and selectively non-ATP competitive. However, it has not received FDA approval due to notable toxicities, such as fatigue, dermatitis acneiform, increased blood CPK, diarrhea, and stomatitis, along with partial anticancer activity [17,18]. Despite extensive efforts spanning over two decades, several targets, including protein kinases, have faced restrictions in gaining FDA approval for their drugs. Additionally, there are also formidable challenges in identifying and validating allosteric inhibitors [16].

## 4. Comparison of Type III Inhibitor Targets

The identification of distinctive structural features in a site (allosteric) adjacent to the typical active site pocket of MEK1/2 has led to the discovery of new drugs for inhibiting MEK1/2, which could potentially be utilized for targeting other kinases as well [19]. Zhao et al. published a comprehensive analysis focusing on the binding mode of cobimetinib, a type III kinase inhibitor, particularly examining its interactions with MEK1/2 proteins [20]. These investigations offer insights into how specific structural characteristics within kinase or catalytic domains can be utilized to design and develop more potent and selective inhibitors. By comparing few existing structures of MEK1 bound to type III inhibitors, the authors acknowledged three distinct allosteric clefts within the site of catalysis, serving as structural hotspots for the development of inhibitors [19]. In our review, we have extended the analysis to include binding pockets of several protein kinases, such as MEK1, Akt, LIMK2, and EGFR, as crucial targets for allosteric inhibitors. This comparison aims to unveil potential structural similarities among them as well as their co-crystallized ligands, which could expedite the development of more selective inhibitors.

### 4.1. Binding Pocket Analysis

#### 4.1.1. SiteMap

The range of methods for comparing binding sites is still expanding, making it increasingly difficult to choose the appropriate method for a particular research topic. Here, we utilize the SiteMap package available in Schrödinger Maestro 13.7 (Release 2023-3, Schrödinger, LLC, New York, NY, USA) [21,22]. SiteMap employs an innovative search and analysis method to provide insights into the characteristics of binding sites. The process begins with an initial search stage that identifies multiple regions near the protein surface, called sites, potentially suitable for ligand binding. Subsequently, site maps are produced, encompassing hydrophilic and hydrophobic features, including acceptor, donor, and metal-binding regions. The evaluation phase, concluding the calculation, scrutinizes each respective site by computing different properties, such as the volume of the binding pocket. Site maps have the potential to enhance the design of ligands by identifying “targets of opportunity”, such as hydrophobic areas capable of accommodating larger hydrophobic groups [23].

These targets might include hydrophobic areas offering ample space for accommodating bigger hydrophobic groups, thereby offering possible avenues for optimizing ligands. Moreover, regions lacking distinct hydrophilic or hydrophobic features are noteworthy, indicating regions where enhancements in the physical properties of the ligand, such as solubility, could be improved, which could have a lesser impact on binding affinity. Unlike approaches utilizing color-coded depictions of hydrophilicity and hydrophobicity on the surface of receptor, site maps take into account the inclusive nature of the site, instead of exclusively focusing on the closest receptor atom. Furthermore, site maps illustrate the boundaries and shapes of both hydrophobic and hydrophilic regions, providing precise detail beyond what surface-based methods can achieve. We needed to explore whether the binding site similarities could account for the occurrence of adequate binding pocket areas for the design of allosteric inhibitors. For further comprehension, the volume of the binding pocket was computed for the selected structures (complex), followed by a subsequent comparison. This analysis intended to elucidate the structural characteristics of ligands and the significant interactions they form.

#### 4.1.2. Comparison of Protein Binding Site

An essential factor in deciphering the biological functions of proteins is their three-dimensional structure. The examination of protein structures, particularly the evaluation of binding sites, is pivotal in the field of drug discovery. Grasping the attributes of binding sites constitutes a fundamental stride in structure-based drug design. Numerous proteins harbor analogous binding sites, and comparisons of these sites offer valuable insights for repurposing existing drugs, identifying potential off-target effects, and establishing polypharmacology [24]. Considering the same phenomena, we decided to compare the binding pockets of protein kinases as targets for potential allosteric inhibitors.

SiteMap was used for the same purpose. Numerous protein kinase structures, co-crystallized with their corresponding allosteric compounds, are accessible on the Protein Data Bank (PDB). To authenticate the aforementioned hypothesis, these kinases were chosen and their binding pockets were compared while taking volume variation, important interactions, and ligand shape into consideration. We compared the binding pockets of a few kinases such as MEK1, AKT, LIMK2, and EGFR, which are listed below in Table 1.

##### Binding Mode of Trametinib and Cobimetinib Inside the Allosteric Pocket of MEK1

**Figure 2:**
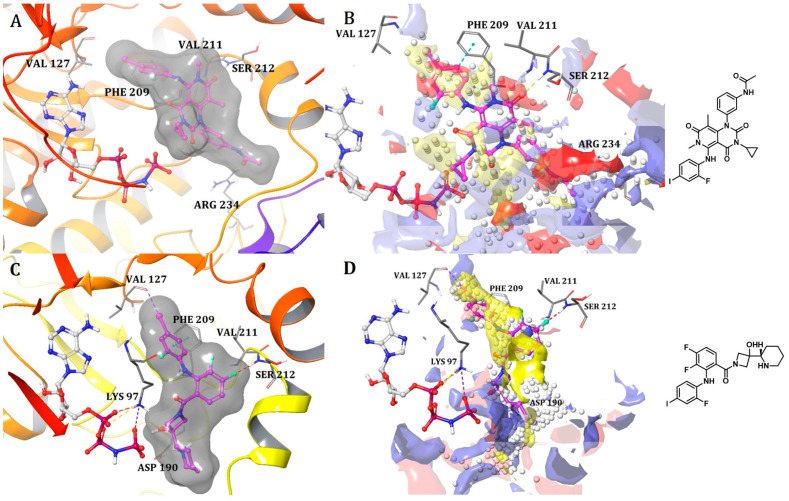
(**A**) Binding mode of trametinib and (**C**) cobimetinib inside the allosteric pocket of MEK1. Ligand structures are shown as transparent surfaces in gray color and sticks are represented by dark magenta color; (**B**) Binding modes of trametinib and (**D**) cobimetinib overlap with site maps in red, blue, and yellow colors that represent hydrogen bond acceptor, hydrogen bond donor, and hydrophobic regions, respectively. Big white dots reveal the available pocket area inside the binding pocket.

The crystal structures of the KSR1:MEK1 complex bound with trametinib (PDB: 7JUX) and MEK1 bound to cobimetinib (PDB: 4LMN) were imported from the Protein Data Bank (PDB) [25,26]. Trametinib is situated within the conventional allosteric site of MEK adjacent to ATP, aligning with its classification as an ATP non-competitive protein kinase inhibitor (Figure 2A). Additionally, trametinib interacts with a protracted sub-pocket that extends to the interaction interface of KSR.

Trametinib can be split into three fragments. The first fragment, containing the 2-fluoro, 4-iodo substituted phenyl moiety, is positioned amidst Met143 and Lys97, which are gatekeeper and conserved residues of subdomain II, respectively, and numerous residues from helix αC (Leu118) and the initial residue, Val127 from β-strand 4 in MEK1. Notably, Val127 plays a critical role in forming a halogen bond. The second fragment resides on one side, adjacent to the N-terminal region of the activation segment, encompassing the DFG motif, commencing at Asp208.

This segment of the inhibitor also establishes hydrogen bonds with the backbone amide of Ser212 and Val211, crucial interactions observed in several other MEK inhibitors. On the opposite side of fragment 2, comprising the cyclopropyl ring, it is situated directly next to the phosphates of ATP. A distinctive feature of trametinib, absent in other clinical MEK inhibitors, is the 3-substituted phenyl acetamide moiety, referred to as fragment 3. The pocket at the interface between MEK and KSR creates room for the accommodation of this fragment, leading to the formation of interactions with MEK’s activation loop through Leu215, Met219, and Ile216. Moreover, the HRD motif is implicated, encompassing Arg189 and Asp190, followed by a hydrogen bond with Arg234 situated toward the end of the activation loop (Figure 2A).

In contrast to trametinib, KSR1 and KSR2 do not engage in direct interactions with the other MEK1 inhibitors, indicating that direct engagement with KSR is a unique characteristic of trametinib. The structural comparisons suggest significant differences in the MEK allosteric pocket between the isolated MEK and a state in which it is bound to KSR. In the isolated MEK state, the activation loop adopts an inward conformation, whereas extended conformation indicates a state bound to KSR [25]. Furthermore, in the case of a KSR-bound complex, trametinib occupies an expanded allosteric pocket in MEK, which is facilitated by direct interactions with KSR [25]. This difference may account for the higher volume of the binding pocket observed in the trametinib-bound MEK1:KSR1 complex compared to cobimetinib, which binds only to MEK1 (Table 1). Additionally, the piperidine moiety of cobimetinib forms a hydrogen bond with Asp190. Although both ligands have similar shapes, they exhibit distinct interactions within the allosteric pocket, which must be considered when developing analogs or more potent MEK1 inhibitors. Figure 2B,D illustrate site maps overlaid with the binding modes of trametinib and cobimetinib, respectively. The site maps, color-coded in blue, red, and yellow to signify hydrogen bond donor, hydrogen bond acceptor, and hydrophobic regions, respectively, aid in understanding the discussed interactions and important hotspots of MEK1, which are crucial for modifying ligand structure to achieve better binding. 

##### Binding Mode of ARQ092 and Borussertib Inside the Allosteric Pocket of AKT1

**Figure 3:**
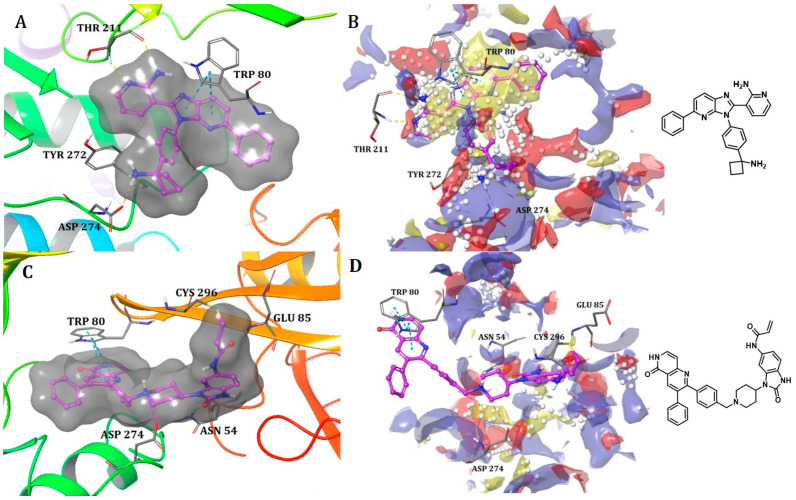
(**A**) Binding mode of ARQ092 and (**C**) borussertib inside the allosteric pocket of AKT1. Ligand structures are shown as transparent surfaces in gray color and sticks are represented by dark magenta color. (**B**) Binding modes of ARQ092 and (**D**) borussertib overlap with site maps in red, blue, and yellow colors that represent hydrogen bond acceptor, hydrogen bond donor, and hydrophobic regions, respectively. Big white dots reveal the available pocket area inside the binding pocket.

The available crystal structures of AKT1 in conjunction with ARQ092 (PDB: 5KCV) [27] and borussertib (PDB: 6HHF) [28] revealed that ARQ092 occupies an allosteric pocket situated at the edge amongst the kinase and PH domain. The aminopyridine group of the (3-phenyl-3H-imidazo[4,5-b]pyridin-2-yl)-pyridin-2-amine scaffold obtains hydrogen bonds with the cluttered helix αC and the β4 strand (Thr211), while the scaffold establishes a crucial hydrophobic interaction with Trp80 that belongs to the PH domain. Furthermore, the phenyl ring of phenylcyclobutylamine directly engages in hydrophobic contact with Tyr272 within the kinase domain that holds an extremely conserved YRD motif. Moreover, the cyclobutylamine possesses bidentate hydrogen bonds with both the carboxylate group of Asp274 and the key chain of Tyr272. This interaction aids in positioning the cyclobutyl group towards the hydrophobic side chain of Ile84 (Figure 3A).

On the other hand, borussertib functions as a covalent allosteric inhibitor of AKT1, binding to the allosteric pocket and containing a Michael acceptor to form covalent bonds with noncatalytic cysteines. These characteristics combine the advantages of exceptional selectivity from PH domain-dependent allosteric inhibition with the therapeutic benefits of irreversible modification, resulting in increased drug target residence times and enhanced activity. The crystal structure reveals an inactive, autoinhibited conformation wherein the PH domain is folded onto the kinase domain (PH-in conformation) between the N- and C-lobes.

These features merge the benefits of PH domain-dependent allosteric inhibition’s remarkable selectivity with the therapeutic advantages of irreversible modification, leading to better drug target residence time and improved activity. The PDB crystal structure depicts an autoinhibited conformation that is inactive, wherein the PH domain is folded over the kinase domain (referred to as the PH-in conformation) between the N- and C-lobes. This arrangement displaces the regulatory αC-helix while simultaneously shaping an allosteric binding pocket in the middle of these two domains. This difference could explain why the volume of the binding pocket in the borussertib–AKT1 complex is smaller than that in the ARQ092-AKT1 complex (Table 1).

Borussertib interacts with the abovementioned allosteric pocket and forms crucial aromatic pi-pi stacking flanked by the 1,6-naphthyridinone and the indole side chain of Trp80 from the PH domain. Additionally, a salt bridge was observed with Asp274. The acrylamide is prealigned through hydrogen bond interaction between the amide oxygen of the warhead and NH of Glu85, thereby aiding in the establishment of a covalent bond between the thiol side chain of Cys296 and the electrophilic β-carbon (Figure 3C). ARQ092 and borussertib were depicted as superimposed with site maps inside the allosteric pocket of AKT1 (Figure 3B,D).

##### Binding Mode of DDC4002 and JBJ-04-125-02 Inside the Allosteric Pocket of EGFR

**Figure 4:**
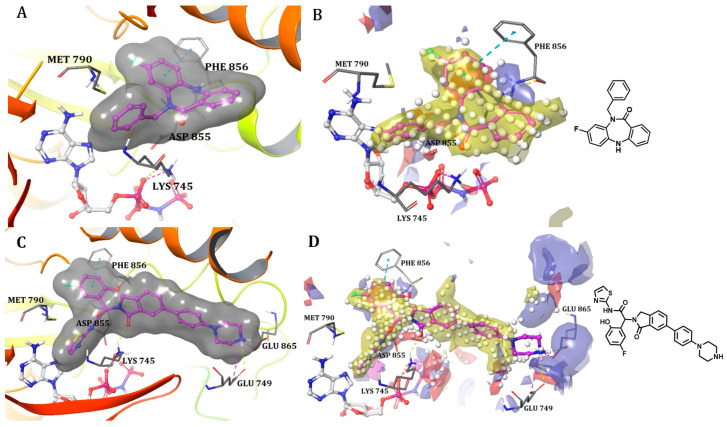
(**A**) Binding mode of DDC4002 and (**C**) JBJ-04-125-02 inside the allosteric pocket of EGFR. Ligand structures are shown as transparent surfaces in gray color and sticks are represented by dark magenta color; (**B**) binding modes of DDC4002 and (**D**) JBJ-04-125-02 overlap with site maps in red, blue, and yellow colors that represent hydrogen bond acceptor, hydrogen bond donor, and hydrophobic regions, respectively. Big white dots reveal the available pocket area inside the binding pocket.

The crystal structures of EGFR (L858R/T790M) in complex with DDC4002 (PDB: 6P1D) [29] and JBJ-04-125-02 (PDB: 6DUK) were retrieved from the PDB [30]. Both inhibitors bind to the kinase domain within an allosteric pocket next to the ATP-binding site. The diazepinone ring of DDC4002 is flexed in the direction of the αC-helix, the 8-fluorobenzene ring is situated inside the hydrophobic back pocket, and the benzene ring, which is unsubstituted, is positioned outwardly towards the solvent. The benzyl moiety prolongs towards the N-lobe of kinase, burrowed between AMPPNP and the side chains of Lys745, Leu788, and the gatekeeper mutation residue, T790M. However, the inhibitor primarily establishes hydrophobic contacts, and the diazepinone NH acquires a hydrogen bond as well as pi-pi stacking with the backbone carbonyl of Phe856, one of crucial residues from the DFG motif (Figure 4A).

The crystal structure (PDB: 6DUK) of JBJ-04-125-02 with T790M-mutant EGFR shows that it binds to the allosteric pocket of EGFR, which is formed by the outward conformation of helix αC. This conformation is in the inactive form (Figure 4C). Furthermore, the carbonyl of Phe856 from the DFG motif is responsible for building H-bonds with hydroxyl groups of JBJ-04-125-02. The 4-piperazinophenyl group is laid along with the solvent exposure region, with its phenyl ring forming pi-pi stacking interaction with the kinase P-loop (Phe723). Remarkably, the compound’s binding elicits a distinct conformational change in the activation loop of kinase, apparently upheld by H-bonds between the piperazine and Glu865 within the activation loop. Additionally, Glu749 is positioned to form H-bonds with the piperazine group. Reportedly, the alteration in the activation loop, along with the subsequent H-bond formed with the piperazine group, contributes to the increased potency of this compound in comparison to EAI045 [31]. The overall analysis indicates that the extended moiety, phenylpiperazine, incorporated in JBJ-04-125-02 increases its IC_50_ value and docks well in the allosteric pocket of EGFR due to additional interactions with Glu749 and Glu865. Hence, it also occupies more volume within the allosteric pocket of EGFR, leading us to conclude that the shape or size of the ligand does not necessarily have to meet a specific limit but must be appropriate enough to bind inside the allosteric pocket for inhibition. This observation receives additional support from the sitemaps illustrated in Figure 4B,D, as the red, blue, and yellow sitemaps align precisely with the designated H-bonds and hydrophobic interactions discussed above.

##### Binding Mode of LIMK2 with Its Co-Crystal Ligand

**Figure 5:**
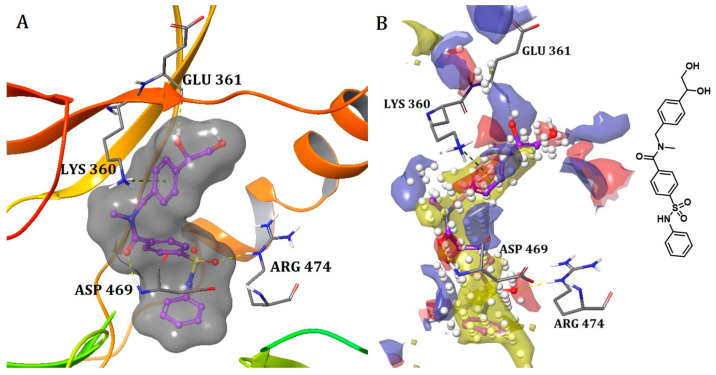
(**A**) Allosteric pocket of LIMK2 with its co-crystal ligand. Ligand structures are shown as transparent surfaces in gray color, and sticks are represented by dark magenta color; (**B**) LIMK2-ligand overlaps with site maps in red, blue, and yellow colors that represent hydrogen bond acceptor, hydrogen bond donor, and hydrophobic regions, respectively. Big white dots reveal the available pocket area inside the binding pocket.

The provided crystal structure (PDB: 4TPT) represents the first type III inhibitor (allosteric inhibitor) of LIM-kinase 2 (LIMK2) [31]. LIM kinases have been thoroughly investigated to determine their role in multiple therapeutic applications, spanning from cancer to open-angle glaucoma. This ligand binds specifically to the hydrophobic pocket created when the residues (DFG) of the activation loop adopt the DFG-out conformation. The amide carbonyl engages with the backbone NH of Asp469 from the DFG residues in the activation loop, elucidating the crucial role of a hydrogen bond acceptor at this location (Figure 5A). Simultaneously, the sulfonamide carbonyl reinforces the ligand by forming a hydrogen bond with the Arg474 residue next to the DFG motif. Both of these hydrogen bonds are supported by the presence of red site maps at the respective positions that indicate favorable positions for H-bond acceptors (Figure 5B). The N-benzylamide moiety extends towards the solvent front and establishes a pi-cation interaction with Lys360. Lastly, the hydroxyl group of phenylethane-1,2-diol forms a hydrogen bond with Glu361. This example exemplifies a compact ligand structure capable of fitting snugly within the small allosteric binding pocket of the kinase (LIMK2) (Table 1 and Figure 5B).

The ligand–protein interaction analysis and calculation of the volume of the binding pocket were conducted for the aforementioned protein kinases obtained from the PDB. These comprehensive observations indicated that despite the significant sequence similarity between two kinases, their binding pocket volumes may not always be identical. The volume of the binding pocket varies depending on factors such as the co-crystallized ligand bound to the protein or the orientation of the p-loop, α-helix, and DGF loop. Furthermore, to design potential allosteric inhibitors for a specific kinase, it is crucial to analyze all previously available inhibitors, including their shape, binding mode, and physicochemical properties. Comparing multiple structures of a target kinase can provide insights into its structural conformations and key mutants. As part of our future endeavors, we may explore the development of allosteric inhibitors for EGFR.

## 5. Conclusions

Our comprehensive review, which involved analyzing existing FDA-approved drugs and crystal structures of other type III inhibitors, indicates that the residues within the allosteric pocket among the selected protein kinases exhibited minimal resemblance to each other. The likelihood of two proteins sharing a similar allosteric pocket in terms of sequence, shape, or size is very low. Therefore, we believe that initially comparing the shapes/structures of ligands and modifying them based on insights gathered from site maps or other QSAR studies would be very helpful. Moreover, to design an allosteric inhibitor targeting a specific kinase, comparing all available structures of the same protein would provide a better understanding of the nature of its allosteric pocket compared to comparing it with other protein kinases.

Allosteric protein kinase inhibitors epitomize a promising novel therapeutic approach for targeting kinases nurturing oncogenic mutations in cancer. Allosteric kinase inhibitors have shown improved characteristics compared with the conventional type I or type II inhibitors and demonstrated potential for developing new drug candidates. In addition to selectivity and potency, structural modifications also have an impact on physicochemical, safety, pharmacodynamic, and pharmacokinetic properties. They represent a valuable approach for the drug design community in the development of novel therapeutic and diagnostic agents for human diseases.

## Figures and Tables

**Table 1:** Two-dimensional structures of co-crystallized ligands of selected protein kinases and their volume of binding pocket.

Number	Protein	PDB ID	2D Structure of Co-Crystallized Ligand	Volume of Binding Pocket (Å^3^)
1	MEK1	7JUX	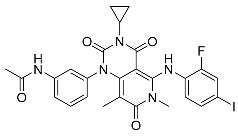 Trametinib *	994.70
		4LMN	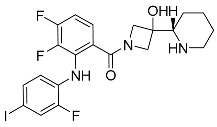 Cobimetinib *	548.11
2	AKT1	5KCV	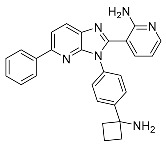 ARQ092	692.17
		6HHF	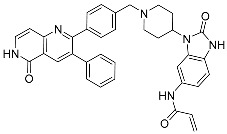 Borussertib	418.11
3	EGFR	6P1D	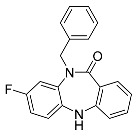 DDC4002	173.21
		6DUK	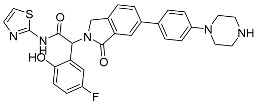 JBJ-04-125-02	416.40
4	LIMK2	4TPT	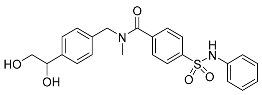 4TPT-ligand	311.44

* FDA-approved drugs.
